# The Story of the Insane from Year to Year

**Published:** 1897-10-09

**Authors:** 


### THE STORY OF THE INSANE FROM YEAR TO YEAR.

### Tenth Series.

### GLASGOW ROYAL ASYLUM.

Here the numbers fell from 448 to 436. There were 92 first admissions, 20 readmissions, 41 recoveries, 52 discharged relieved, and 31 deaths. The recoveries stand at 36·6 per cent. on the admissions, and the deaths at 5·5 on the average number resident. Of the deaths 16 were caused by cerebral and spinal affections, and 5 by consumption. Twenty of the year's admissions owed their insanity to domestic trouble and other moral causes, 16 to intemperance in drink, and 8 to hereditary tendency. Dr. Yellowlees shows that the diminution in the numbers has nothing to do with the increase or decrease of insanity, and that such questions can only be tested by comparing a definite area in corresponding periods, and he says that "such comparisons lead to the conclusion that there is little or no real increase." We are pleased to have this expression of belief from so eminent an authority, and while giving due weight to it, we fear there is in some districts at least an actual increase of insanity greater than can be fairly accounted for by the general increase of the population and the mere accumulation of lunatics. Dr. Yellowlees sums up the prospect of recovery from insanity so well that we cannot refrain from quoting the paragraph. Omitting imbecility and senility, he says that "Of 12 persons attacked by insanity one half will recover and the other half will not; of the 6 who recover one half will remain permanently well, while the other half will become insane again once or oftener in the course of their lives, and will eventually die insane." When we are told that 193 of the patients pay less than £40 per annum we do not require any better evidence of the good work this asylum is doing, and we are also told that "no effort is spared by Dr. Yellowlees and his medical staff to secure the recovery of the curable and the comfort and well-being of the incurable."

### ISLE OF WIGHT ASYLUM AT NEWPORT.

This (the first annual) report tells us that there were 236 patients in residence on the last day of 1896, and that 214 had been transferred from other asylums, the majority being from the Hants County Asylum. As the asylum was only opened in September, we need not quote the numbers admitted, discharged, and died, as no good end could thereby be served. The contractors began their work in February, 1894, and, as already said, the asylum was opened in September, 1896, so that it must have been very expeditiously built. Dr. Shaw says the site is a very favourable one, and the buildings of broken contour and diversified appearance, and will lend themselves admirably to decoration by the hand of time. The asylum will contain 260 pauper patients, and there is a detached block for 50 private cases; but as there are at present only 22 vacancies for paupers, it is evident that the asylum will have to be enlarged very soon. The visitors say that high "credit reflects on the medical superintendent for his able management."

### THE MIDDLESEX COUNTY ASYLUM AT WANDSWORTH.

An increase from 1,170 to 1,235 has to be noted during the year 1896. There were 289 first admissions, 51 readmissions, 126 discharged recovered, 49 discharged relieved, and 105 deaths. The recoveries stand at 39·4 per cent. of the admissions, and the deaths at 8·7 per cent. of the average number resident. Of the deaths 41 were caused by cerebral and spinal diseases, and 15 by phthisis. Of the admissions 48 were believed to be caused by various moral causes, 33 by intemperance in drink, and 75 by hereditary influence. Dr. Hill points out that further accommodation will soon be required on the female side of the asylum. There are at present 804 patients requiring accommodation and only 687 beds, but when the new buildings are finished there will be room for 787. The asylum was visited during the year by scarlet fever, and 14 patients and two attendants were attacked. All recovered. We are glad to be able to congratulate Miss Hawkins, the assistant head nurse, on having obtained her pension after 18 years' service. A block is about to be opened for the treatment of idiot and imbecile children. There is no doubt that much may be done in the way of educating and training this class, and we agree with Dr. Hill when he says "it would be a noble achievement to bring back this unfortunate class within the pale of ordinary life," and we wish him every success in his undertaking. The committee express their indebtedness to Dr. Hill and the staff generally for the thoroughness with which they have discharged their duties. The weekly cost per head is 11s. 2d.

